# Impacts of iodoacetic acid on reproduction: current evidence, underlying mechanisms, and future research directions

**DOI:** 10.3389/fpubh.2024.1434054

**Published:** 2024-10-03

**Authors:** Mei Ha, Li Mou, Jiayuan Qu, Changjiang Liu

**Affiliations:** ^1^Chongqing Medical and Pharmaceutical College, Chongqing, China; ^2^NHC Key Laboratory of Birth Defects and Reproductive Health, Chongqing Population and Family Planning Science and Technology Research Institute, Chongqing, China

**Keywords:** drinking water disinfection byproduct, fertility, iodoacetic acid, reproductive damage, reproductive toxicant

## Abstract

In light of the undeniable and alarming fact that human fertility is declining, the harmful factors affecting reproductive health are garnering more and more attention. Iodoacetic acid (IAA), an emerging unregulated drinking water disinfection byproduct, derives from chlorine disinfection and is frequently detected in the environment and biological samples. Humans are ubiquitously exposed to IAA daily mainly through drinking water, consuming food and beverages made from disinfected water, contacting swimming pools and bath water, etc. Mounting evidence has indicated that IAA could act as a reproductive toxicant and bring about multifarious adverse reproductive damage. For instance, it can interfere with gonadal development, weaken ovarian function, impair sperm motility, trigger DNA damage to germ cells, perturb steroidogenesis, etc. The underlying mechanisms predominantly include cytotoxic and genotoxic effects on germ cells, disturbance of the hypothalamic–pituitary-gonadal axis, oxidative stress, inhibition of steroidogenic proteins or enzymes, and dysbiosis of gut microbiota. Nevertheless, there are still some knowledge gaps and limitations in studying the potential impact of IAA on reproduction, which urgently need to be addressed in the future. We suppose that necessary population epidemiological studies, more sensitive detection methods for internal exposure, and mechanism-based in-depth exploration will contribute to a more comprehensive understanding of characteristics and biological effects of IAA, thus providing an important scientific basis for revising sanitary standards for drinking water quality.

## 1 Introduction

Accumulating evidence has suggested that human fertility has gradually declined in recent decades, and it is estimated that approximately 186 million couples of reproductive age worldwide are influenced by infertility (1). Therefore, to identify the potential risk factors contributing to the decline in human fertility has become a significant public and clinical health concern. Apart from genetic factors, a growing body of population and experimental research has demonstrated that environmental factors, especially exposure to environmental pollutants, play a prominent role in unfavorable reproductive outcomes, such as infertility, abnormal pregnancy, miscarriage, preterm delivery, disorder of sex hormone levels, sexual dysfunction, etc. (2–6).

Drinking water disinfection is an effective means to eliminate waterborne diseases and is regarded as one of the most significant and practical public health achievements of the 20th century. In the disinfection process, however, disinfection byproducts (DBPs) are unintentionally produced from the reaction of highly reactive disinfectants with organic or inorganic components including bromide, chloride, and iodide in raw water (7). For instance, iodinated DBPs (I-DBPs) are formed during the disinfection of iodide-containing raw water. The unavoidable generation of DBPs in drinking water has aroused public concern for health. In particular, a large number of studies have linked DBPs exposure to various reproductive impairments and even infertility, such as steroidogenesis suppression, spermatogenetic dysfunction, diminished ovarian reserve (8–11). So far, over 700 DBPs have been discovered in drinking water, but only 11 of them are currently under supervision of regulatory agencies. Actually, an increasing body of literature has revealed that some hazardous health effects mediated by DBPs, including reproductive damage, are often attributed to unregulated DBPs, which in many cases exhibit greater toxicity or toxic potential than regulated DBPs (12, 13).

**Figure 1:**
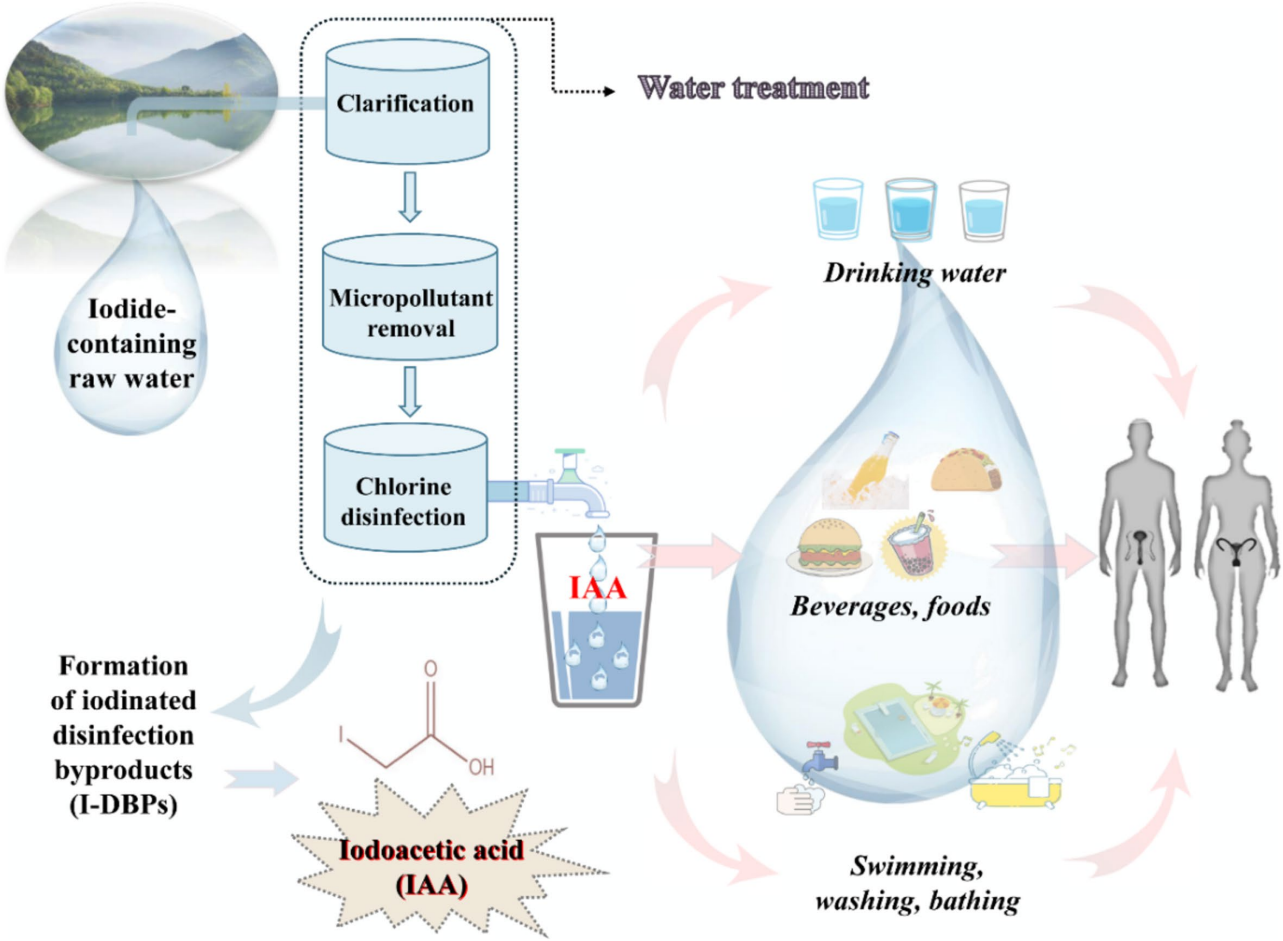
A schematic model demonstrated the underlying exposure routes of iodoacetic acid (IAA, an iodinated disinfection byproduct of drinking water).

Iodoacetic acid (IAA) is an emerging unregulated iodinated DBP, which has attracted considerable attention in recent years due to its higher cytotoxicity and genotoxicity than other DBPs (14, 15). A large quantity of environmental monitoring data have shown that IAA is frequently detected in drinking water, and its concentration is up to 1.7 μg/L in the United States and Canada (16) and up to 2.18 μg/L in China (17). In coastal and inland regions with high iodine levels, the IAA concentration in drinking water may be higher with the increase in iodide contents in raw water. Humans are ubiquitously exposed to IAA on a daily basis, primarily through drinking water, consuming food and beverages made with disinfected water, and contact with swimming pools and bath water, among other sources (Figure 1). Therefore, given the high toxicity and persistent exposure to IAA, it is crucial to comprehensively evaluate its toxicity and detrimental health effects on organisms. In recent years, a series of adverse health effects of IAA have been gradually observed and reported, such as the carcinogenic effect (17), cytotoxic effect (18, 19), thyroid dysfunction (20, 21), gut microbiota disorder (22), developmental disturbance (23), retinal degeneration (24, 25), etc. Although direct evidence from population research is scarce, several *in vivo* and *in vitro* studies have proposed that IAA could act as a reproductive toxicant and potentially interfere with reproduction and fertility. For instance, our latest work uncovered that IAA possessed an anti-androgenic property and impeded testosterone biosynthesis in Leydig cells through the crosstalk between the GRP78/IRE1/XBP1 pathway and the cGAS/STING pathway (26). Hence, this paper reviewed the limited literature on IAA exposure in humans, animals, and cells to figure out the negative effect of IAA on reproduction, and further explored its underlying mechanisms of action. This review will facilitate a better understanding of deleterious effects of IAA on reproductive health and a more comprehensive assessment of toxic effects and biological features of IAA, thus providing an important scientific basis for revising sanitary standards for drinking water quality.

## 2 Methodology

The search databases were Web of Science, PubMed, and Google Scholar. The retrieval time node ranged from Jan. 2000 to Jul. 2024. The retrieval strategy was optimized with the use of Boolean logical operators. The search terms included, but were not limited to, disinfection byproducts, DBPs, iodinated DBPs, I-DBPs, iodoacetic acid, IAA, reproduction, reproductive impairment, fertility, infertility, testis, prostate, epididymis, sperm, ovary, uterus, follicle, and steroid hormone. The inclusion criteria were as follows: (a) studies investigated the deleterious effects of IAA on reproduction; (b) studies were performed in humans, animals, or cells; (c) studies were available as full-text publications; and (d) studies were presented in English. Finally, a total of 98 studies were identified and 53 were included in this review.

## 3 IAA-induced harmful effects on reproductive health

After exposure to IAA, it could enter the body and accumulate in diverse tissues and organs, including reproductive organs. For instance, in a recent study on the internal exposure level of IAA, after oral exposure to 6 mg/kg of IAA, it was detected in rat testes and its concentration reached up to 1.12 ng/g (27). Therefore, long-term exposure to IAA, an emerging endocrine disruptor, is likely to bring a series of reproductive disorders to both humans and animals.

### 3.1 Hazardous effects of IAA on male reproductive health

The research by Ali et al. (28) was performed to evaluate the direct influence of IAA exposure on sperm using semen samples provided by healthy volunteers. After treatment with 25 μM of IAA for 1 h, apparent sperm DNA damage and genotoxic responses were observed using the comet assay. However, these harmful effects caused by IAA could be effectively antagonized and alleviated by the antioxidant butylated hydroxyanisole (BHA) or catalase. Therefore, it is supposed that the redox imbalance may be an important mode of action of IAA-triggered unfavorable effects on reproduction. Similar reproductive damage effects also appeared in an IAA-treated male mouse model. After exposure to 35 mg/kg of IAA for consecutive 28 days via oral gavage, IAA not only lowered the sperm motility by directly reducing the average path velocity (VAP) and straight line velocity (VSL) of sperm, but also brought about DNA double-strand breaks of spermatogenic cells, especially that of spermatocytes (9). Moreover, IAA also interfered with steroidogenesis, giving rise to an increase in serum LH levels and a borderline decrease in testosterone levels. SRB1 is responsible for the transport of high-density lipoprotein (HDL) cholesteryl esters into Leydig cells (29), and the inhibition of SRB1 protein expression could partially account for the declining trend of serum testosterone levels after IAA exposure (9). Similar results were also observed in our recent rat model. According to the previous investigation by Xia et al. (20), the acute oral LD50 value of IAA in male rats was 126 mg/kg. After exposure to 25 mg/kg (1/5 of LD50) of IAA for 31 days, the testicular histomorphology and ultrastructure of exposed rats were abnormally changed and testosterone biosynthesis was inhibited, followed by a decrease in the testosterone level in rat plasma (26). In reproductive/developmental toxicity screening tests, SD rats were treated with 22.5 mg/kg of IAA for consecutive 28 days and the sperm motility was declined in a dose-dependent manner. In addition, relative weights of testes and seminal vesicles plus coagulating glands (SVCG) in parental male rats were increased, while the anogenital distance (AGD) index of male pups was decreased after IAA exposure (23). These research findings are considered to be associated with the androgen disturbing characteristic of IAA. This perspective is supported by another study conducted in male rats. Sha et al. (22) also proposed that IAA could act as an androgen disruptor. After exposure to 16 mg/kg of IAA for consecutive 56 days, IAA perturbed the abundance of steroid hormone biosynthesis-related genes in the gut microbiota of male rats via the dysbiosis of intestinal flora and its metabolism, finally influencing androgen levels of male rats.

In conclusion, though employing different exposure models, routes, dosages, and periods, above evidence from populations and animals has clearly indicated that IAA has the interference effect on male reproductive health. IAA could influence gonadal development, facilitate sperm DNA damage, impair testicular histomorphology and ultrastructure, decline sperm motility, and perturb the biosynthesis of male steroid hormones (Table 1). Nevertheless, it should be noted that though some progress has been made, most of the evidence comes from the experimental models, and the evidence from population studies is relatively insufficient. Moreover, there are still knowledge gaps that merit further research. For instance, could IAA interfere with the microenvironment of sperm production by affecting the blood-testis barrier? Could IAA disturb feedback regulation of the hypothalamic–pituitary-testis (HPT) axis by regulating hormone receptor binding? Without doubt, more population epidemiological research and further mechanism-based studies are needed in the future to address these pending concerns.

**Table 1:** Toxicological studies on IAA exposure and male reproductive toxicity.

References	Models	Routes of exposure	Dose/concentration of exposure	Duration of exposure	Observed reproductive effects
Ali et al. (28)	Semen samples from healthy volunteers	Via the culture medium	25 μM	1 h	1. Sperm DNA damage; 2. Display genotoxic responses; 3. Trigger redox imbalance.
Liang et al. (9)	C57BL/6 mice	Via the oral gavage	35 mg/kg	28 days	1. Increase LH levels and borderline decrease testosterone levels; 2. Reduce the average path velocity (VAP) and straight line velocity (VSL) of sperm; 3. Cause DNA damage of spermatogenic cells; 4. Inhibit SRB1 protein expressions in Leydig cells.
Long et al. (23)	Male SD rats	Via the oral gavage	2.5, 7.5, and 22.5 mg/kg	28 days	1. Increase relative weights of testes and seminal vesicles plus coagulating glands in parental male rats; 2. Decrease the anogenital distance index of male pups; 3. Decline the sperm motility in a dose-dependent manner.
Mou et al. (26)	Male SD rats	Via the oral gavage	6.25, 12.5, and 25 mg/kg	31 days	1. Increase relative weights of testes and damage testicular histomorphology and ultrastructure; 2. Reduce Leydig cell numbers and inhibit cell growth; 3. Suppress testosterone production and reduce its levels.
Sha et al. (22)	Male SD rats	Via the oral gavage	16 mg/kg	56 days	1. Act as an androgen disruptor; 2. Upregulate the steroid hormone biosynthesis-related gene abundance in the gut microbiota of male rats; 3. Perturb gut microbiota and metabolism.

### 3.2 Hazardous effects of IAA on female reproductive health

Compared with studies on male reproduction, research on the detrimental impact of IAA on female reproductive health is more abundant. The study by Xia et al. (20) revealed that after exposure to 24 mg/kg of IAA for consecutive 28 days through oral gavage, the ovarian weight of female SD rats was significantly downregulated, while the hypothalamic weight was upregulated. Intriguingly, IAA had different effects on the gonadal development of female offspring. During prenatal, gestational, and lactational exposure to 500 mg/L of IAA, it reduced the vaginal opening rate and the percentage of atretic follicles of female pups, but elevated the absolute weight of ovaries and the anogenital index. Moreover, IAA also gave rise to a borderline decline in progesterone and FSH levels and an increase in testosterone levels in female pups (30). In addition, there is evidence that the estradiol level is also a potential disturbing target for IAA. After exposure to IAA for consecutive 35 days, IAA significantly decreased estradiol levels in female CD-1 mice. Besides, IAA not only shortened the estrous cyclicity, but also disturbed the mRNA level of ovarian genes (31, 32). Gonzalez et al. (33) proposed that IAA-mediated female reproductive impairment was closely associated with the disturbance of the hypothalamic–pituitary-ovary (HPO) axis, as evidenced by an increase in kisspeptin mRNA levels in the hypothalamic arcuate nucleus and a decrease in FSHβ-positive cell numbers and FSHβ mRNA levels in the pituitary in female CD-1 mice. What’s more, IAA led to DNA damage and induced P21/Cdknɑ mRNA levels in the pituitary in female mice. Likewise, gut microbiota dysbiosis also played an important role in IAA-aroused abnormality of hormone levels in females. It was reported that IAA exposure for 8 weeks led to an upregulation of androstanediol levels in female SD rats by disturbing gut microbiota (22). Furthermore, growing *in vitro* studies indicate that IAA has potent cytotoxic and genotoxic effects on ovarian cells, thereby interfering with the growth, maturation, and function of ovaries. This is evidenced by a series of studies carried out in Chinese hamster ovary (CHO) cells (34–36). IAA treatment not only increased the mutant frequency (MF) in ovarian cells, but also promoted the damage to genomic DNA and lowered the rate of DNA repair of ovarian cells (37, 38), which were partially attributed to oxidative stress. Because the antioxidant BHA or catalase could substantially reverse IAA-mediated genomic DNA damage of CHO cells (37). These findings are in agreement with that of a subsequent investigation. IAA treatment promoted reactive oxygen species (ROS) production and mitochondrial stress, and then contributed to genomic DNA damage as well as the suppression of cellular GAPDH, ATP, and pyruvate levels in ovarian cells (39, 40). Apart from DNA damage, Jiao et al. (41) uncovered IAA could disrupt mouse oocyte maturation by triggering abnormal spindle assembly and chromosome misalignment and causing the metaphase I arrest. In addition, several studies discovered that IAA not only restrained antral follicle growth and proliferation, estrogen receptor *α* (ERα) levels, and steroidogenesis, but also declined estradiol levels in mouse ovarian follicles (15, 42). In terms of underlying effects of IAA on estradiol and ERα levels, however, there are inconsistent findings. In human choriocarcinoma placental (JEG-3) cells, IAA exerted an estrogenic effect and then promoted estradiol levels in JEG-3 cells (43). Another study uncovered that IAA regulated ERα expressions in a species-specific manner. Specifically speaking, IAA showed the robust estrogenic activity on human ERα (hERα), whereas strongly refrained the estrogenic activity on zebrafish ERα (zERα) (44). We speculate that the specificity of species and cells may be the dominant reason for this disparity. Additionally, the difference in IAA concentrations used in these studies would also account for the inconsistence in part. For instance, the IAA concentration used in the study by Mestres et al. (43) was 0.01 to 0.5 μM, while that in the research by Jeong et al. (15) was 2 to 15 μM. Maybe, there exists a low-dose stimulant effect, which deserves further study.

In summary, a mass of toxicological evidence has convincingly demonstrated that IAA has the adverse effect on female reproductive health. IAA could postpone gonadal development, exert cytotoxic and genotoxic effects on ovarian cells, disrupt oocyte maturation, bring about genomic DNA damage, impede DNA repair, affect steroid hormone biosynthesis, etc. (Table 2). Similarly, it should be noted that there is a lack of population epidemiological research in the current evidence. Therefore, it is quite necessary to carry out some cohort studies in the future to search for direct evidence, which could reveal the reliable association between IAA exposure and female reproductive outcomes.

**Table 2:** Toxicological studies on IAA exposure and female reproductive toxicity.

References	Models	Routes of exposure	Dose/concentration of exposure	Duration of exposure	Observed reproductive effects
Xia et al. (20)	Female SD rats	Via the oral gavage	6, 12, and 24 mg/kg	28 days	Decrease the ovarian weight but increase the hypothalamic weight.
Gonsioroski et al. (30)	Female CD-1 mice; F1 female offspring	via the drinking water	10, 100, and 500 mg/L	Adult: prenatal exposure for 35 days and continue exposure in gestation and lactation; Offspring: from lacation until PND21.	1. decrease the vaginal opening rate and the percentage of atretic follicles, while increase the absolute weight of ovaries and the anogenital index in female pups; 2. borderline decrease progesterone and FSH levels, while increase testosterone levels in female pups.
Gonsioroski et al. (32)	Female CD-1 mice	Via the drinking water	0.5, 10, 100, and 500 mg/L	35 days	1. Reduce the estrous cyclicity and estradiol levels; 2. Disturb the mRNA level of ovarian genes.
Gonsioroski et al. (31)	Female CD-1 mice	Via the drinking water	10 and 500 mg/L	35 days	1. Decrease estradiol levels; 2. Disorder mRNA levels in mouse ovarian follicles.
Gonzalez et al. (33)	Female CD-1 mice; pituitary explants from mice	Via the drinking water; the culture medium	0.5, 10, 100, and 500 mg/L; 20 μM	35 days; 48 h	1. Disturb the hypothalamic–pituitary-gonadal axis. 2. Induce kisspeptin mRNA expression in the arcuate nucleus of hypothalami. 3. Reduce FSHβ-positive cell numbers and FSHβ mRNA levels in pituitaries. 4. Induce DNA damage and P21/Cdknɑ mRNA levels in pituitaries.
Sha et al. (22)	Female SD rats	Via the oral gavage	16 mg/kg	56 days	1. Act as an androgen disruptor and increase androstanediol levels in female rats; 2. Interfere with gut microbiota and metabolism.
Plewa et al. (35)	Chinese hamster ovary (CHO) cells	Via the culture medium	10 μM - 1 mM	72 h	Be the most cytotoxic and genotoxic DBPs in ovarian cells.
Plewa et al. (34)	Chinese hamster ovary (CHO) cells	Via the culture medium	10 μM	72 h	Have strong cytotoxic and genotoxic effects on ovarian cells.
Zhang et al. (36)	Chinese hamster ovary K1 (CHO-K1) cells	Via the culture medium	1–27 μM	72 h	1. Induce chronic cytotoxicity; 2. Increase the mutant frequency (MF) in ovarian cells.
Cemeli et al. (37)	Chinese hamster ovary (CHO) cells	Via the culture medium	0.5–25 μM	72 h	1. Exert cytotoxic and genotoxic effects on ovarian cells; 2. Trigger oxidative stress and DNA damage.
Komaki et al. (38)	Chinese hamster ovary (CHO) cells	Via the culture medium	25 μM	24 h	Exert genotoxic effects by mediating the genomic DNA damage and lowering the rate of DNA repair of ovarian cells.
Dad et al. (39)	Chinese hamster ovary (CHO) cells	Via the culture medium	25 and 40 μM	4 h	1. Inhibit cellular GAPDH, ATP, and pyruvate levels. 2. Produce ROS and mitochondrial stress. 3. Induce genomic DNA damage.
Dad et al. (40)	Chinese hamster ovary (CHO) cells	Via the culture medium	3 μM	4 h	1. Reduce cellular GAPDH and ATP levels in ovarian cells. 2. Disturb metabolism of ovarian cells.
Jiao et al. (41)	Oocytes isolated from female CD-1 mice	Via the culture medium	2, 5, and 10 μM	2 and 14 h	1. Disrupt mouse oocyte maturation; 2. Trigger the abnormal spindle assembly and chromosome misalignment; 3. Induce DNA damage; 4. Cause the metaphase I arrest.
Gonsioroski et al. (42)	Antral follicles isolated from female CD-1 mice	Via the culture medium	2, 5, 10, and 15 μM	96 h	1. Inhibit the follicle growth, cell proliferation, and estrogen receptor *α* (ERα) levels; 2. Interfere with steroidogenesis in mouse ovarian follicles.
Jeong et al. (15)	Antral follicles isolated from female CD-1 mice	Via the culture medium	2, 5, 10, and 15 μM	24, 48, and 96 h	1. Inhibit antral follicle growth and steroidogenesis in mouse ovarian follicles; 2. Reduce estradiol levels.
Mestres et al. (43)	Human choriocarcinoma placental (JEG-3) cells	Via the culture medium	0.01, 0.1 and 0.5 μM	24 h	1. Show an estrogenic effect; 2. Elevate estradiol levels.
Lee et al. (44)	Human embryonic kidney 293 (HEK293) cells	Via the culture medium	0.5–500 μM	24 h	Exert species-specific induction or inhibition effects on ERα expressions.

## 4 Potential mechanisms of IAA-mediated reproductive damage

**Figure 2:**
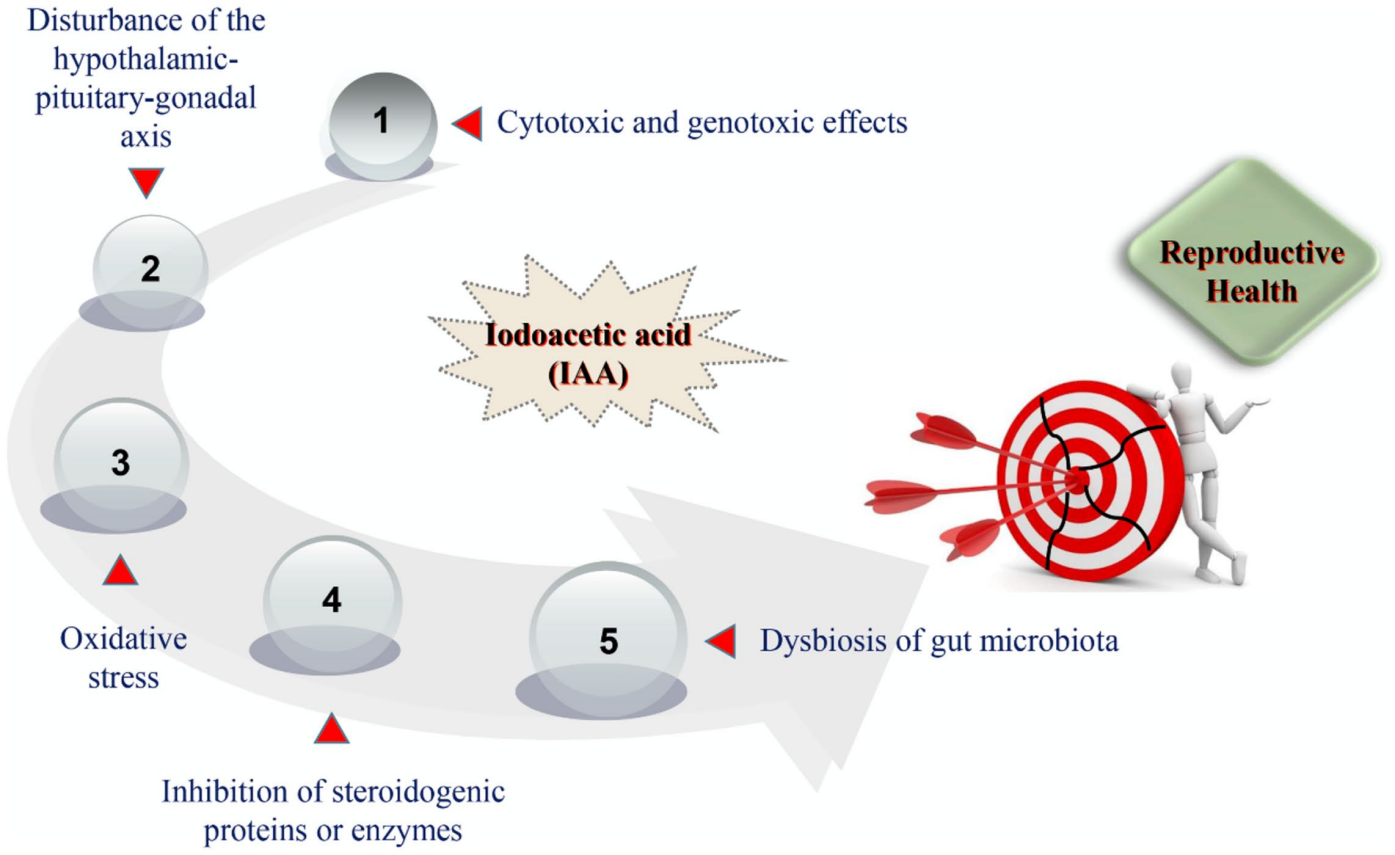
The potential mechanisms of IAA-mediated reproductive damage.

At present, the modes of action by which IAA triggers harmful effects on reproductive health have not been clearly characterized. After reviewing the current toxicological evidence from *in vivo* and *in vitro* studies, we summarized that the potential mechanisms of IAA-mediated reproductive impairment predominantly consisted of cytotoxic and genotoxic effects on germ cells, disturbance of the hypothalamic–pituitary-gonadal (HPG) axis, oxidative stress, inhibition of steroidogenic proteins or enzymes, and dysbiosis of gut microbiota (Figure 2).

### 4.1 Cytotoxic and genotoxic effects

In recent years, IAA has been paid increasing attention because of its potent cytotoxicity and genotoxicity. Previous research has shown that IAA is more cytotoxic and genotoxic than the chlorinated and brominated analogues, such as chloroacetic acid and bromoacetic acid in mammalian cell assays (34). The study on human semen samples revealed that IAA exposure aroused apparently genotoxic and cytotoxic responses and then directly damaged sperm DNA, ultimately impairing semen quality (28). The similar finding was observed in IAA-exposed mice. After four-week exposure, IAA directly brought about DNA damage of spermatogenic cells and then decreased the average path velocity (VAP) and straight line velocity (VSL) of sperms (9). Our recent work has also indicated that the Leydig cell is one of the potential targets of IAA. Following IAA exposure, it not only reduced the amount of Leydig cells in rat testes, but also restrained the cell viability and growth (26). A large number of studies have suggested that IAA could damage female reproductive health by producing strong cytotoxicity and genotoxicity to germ cells. The study by Komaki et al. (38) uncovered that IAA led to the genomic DNA damage and lowered the rate of DNA repair of ovarian cells, thereby weakening ovarian functions. IAA treatment also directly injured oocytes of female mice by its cytotoxic and genotoxic effects, as evidenced by the abnormal spindle assembly and chromosome misalignment, metaphase I arrest, DNA damage, oocyte maturation disorder, etc. (41). Moreover, the antral follicle is also suggested to be the cytotoxic and genotoxic target of IAA, as IAA could restrain antral follicle growth and proliferation (15, 42). Therefore, it is not difficult to see that IAA could give rise to reproductive impairment via the directly cytotoxic and genotoxic effects on germ cells.

### 4.2 Disturbance of the hypothalamic–pituitary-gonadal axis

The hypothalamic–pituitary-gonadal (HPG) axis has pleotropic biological functions and its homeostasis plays an essential role in reproduction and fertility. Literature has indicated that the HPG axis may be one of potential targets of IAA. Xia et al. (20) discovered that IAA could perturb the development of the HPG axis-associated organs and then interfere with its functions. After 4 weeks of exposure to IAA, IAA directly increased the hypothalamic weight while decreased the ovarian weight in female mice. The findings are in agreement with a later study by Gonzalez et al. (33), who also put forward that IAA-caused female reproductive injury was partially attributed to the abnormality of the hypothalamic–pituitary-ovary (HPO) axis. After IAA treatment, the kisspeptin mRNA level in hypothalamic arcuate nuclei was induced, but the FSHβ-positive cell number and FSHβ mRNA level in pituitaries were both declined in female mice, thereby disordering the feedback regulation of the HPO axis. Likewise, in IAA-exposed male mice, the normal feedback regulation of the hypothalamic–pituitary-testis (HPT) axis was also broken down (9). The increase in LH levels secreted by the pituitary failed to stimulate the testis to biosynthesize more testosterone as expected, but instead led to a marginal downregulation. Similarly, the disturbance of the HPT axis was also observed in IAA-treated rats. Mou et al. (26) reported that after IAA exposure, plasma levels of GnRH produced by the hypothalamus and LH produced by the pituitary were not upregulated as expected to respond to the decline in testosterone by IAA. Thus, we suppose that IAA could impair reproductive health by interfering with the development and feedback regulation of the HPG axis.

### 4.3 Oxidative stress

Oxidative stress will happen when the generation of reactive oxygen species (ROS) prevails over the antioxidant capacity of cells. Oxidative stress functions significantly in a series of harmful reproductive outcomes, such as the inhibition of steroid hormone production, decline in semen quality, dysfunction in ovarian function, infertility, etc. (29, 45). Mounting literature from toxicological studies has indicated that oxidative stress contributes to the reproductive impairment mediated by IAA. Due to the insufficient antioxidant capacity and lack of DNA repair mechanisms, sperm is sensitive to and vulnerable to redox imbalance (46). Semen samples from healthy volunteers were treated with 25 μM of IAA for 1 h and the redox homeostasis of spermatids was destroyed, followed by the sperm DNA damage. However, antioxidants including BHA and catalase could effectively antagonize and alleviate oxidative stress-triggered injury effects on sperm (28). Apart from sperms, germ cells are also sensitive to oxidative stress. Following IAA exposure, IAA triggered DNA double-strand breaks of spermatogonia and Leydig cells in rat testes through oxidative stress, as evidenced by an increase in levels of 8-OHdG and *γ*-H2A.X (26). Several *in vitro* investigations also revealed that IAA treatment significantly stimulated ROS production and oxidative stress, subsequently resulting in genomic DNA damage of Chinese hamster ovary (CHO) cells and cell dysfunction (37, 39). Above evidence demonstrates that oxidative stress is one of common and important modes of action by which IAA leads to reproductive damage.

### 4.4 Inhibition of steroidogenic proteins or enzymes

It is known that steroidogenic proteins or enzymes play an essential role in steroid hormone biosynthesis. For instance, testosterone biosynthesis in Leydig cells is exquisitely catalyzed and modulated by a series of steroidogenic proteins or enzymes, such as SRB1, StAR, P450scc, P450c17, etc. Growing evidence has suggested that the steroidogenic proteins or enzymes are the susceptible target of environmental contaminants. The study by Duan et al. (47) disclosed that triclosan exposure inhibited the JAK1/STAT1 pathway via miR-142-5p to limit the transcription and translation of steroidogenic P450c17, thereby restraining testosterone biosynthesis. Another research uncovered that after beta-cypermethrin exposure, overexpressed intronic miR-140-5p negatively regulated steroidogenic StAR, P450scc, and 3β-HSD by targeting SF-1, thereby lowering testosterone levels in rat plasma (48). Likewise, some previous work exhibited that IAA exposure could inhibit steroidogenic protein expressions or enzyme activities. After 28 days of exposure to IAA, IAA depressed SRB1 protein expressions and then reduced the transport of lipid droplet into Leydig cells, leading to a decrease in raw materials for testosterone biosynthesis and a causal decline in testosterone levels in mouse serum (9). This finding is consistent with that of other investigations (22, 26, 42). Hence, these results manifest that IAA could disturb steroid hormone biosynthesis by affecting steroidogenic proteins or enzymes, thereby bringing about detrimental effects on reproductive health.

### 4.5 Dysbiosis of gut microbiota

With a deeper understanding of the gut microbiota, accumulating evidence has shown that the gut microbiota is closely involved in reproductive health and functions significantly through various means, such as gut metabolism, nutrition intake, ROS generation, inflammation (49). The study by Colldén et al. (50) uncovered that the gut microbiota took part in the synthesis and metabolism of androgen and acted as a dominant regulator of androgen metabolism in intestinal contents. Zhang et al. (51) disclosed that the gut microbiota could cross the blood-testis barrier to modulate spermatogenesis, putting forward that the gut microbiota could be employed to treat male infertility by improving the semen quality and spermatogenesis. Toxicological research suggests that exposure to certain DBPs could give rise to dysbiosis of gut microbiota in organisms. The study by Xue et al. (53) discovered that after exposure to dichloroacetamide (DCAcAm), an emerging disinfection byproduct, the gut microbiota composition was disordered in adult zebrafish. Similarly, IAA exposure facilitated gut microbiota dysbiosis, changed the steroid hormone biosynthesis-related gene abundance in the gut microbiota, and perturbed gut microbiota metabolism of male and female rats, ultimately functioning as an androgen disruptor (22). Together, IAA could adversely affect reproductive health through dysbiosis of gut microbiota, which might be considered as a novel mode of action.

## 5 Conclusion and perspectives

Reproductive health is closely related to the sustainable development of mankind and thus, the declining trend in human reproductive health has been an increasing concern all over the world. IAA, as an emerging unregulated iodinated disinfection byproduct, is frequently examined in drinking water and the detected concentration is up to 2.18 μg/L. In recent years, IAA has been considered as an androgen disruptor and may exert an estrogenic effect. Growing *in vivo* and *in vitro* toxicological studies have suggested a possible association between IAA exposure and multiple hazardous effects on reproductive health, such as impairing the sperm motility, disturbing steroidogenesis, triggering DNA damage to germ cells, interfering with oocyte maturation and estrous cyclicity, affecting gonadal development, etc. Although some progress has been made in exploring the impact of IAA on reproductive health, there are still some limitations that need to be further addressed. Firstly, sufficient population epidemiological data are absent from the available evidence. Therefore, it is quite meaningful to carry out necessary population cohort studies to reveal the correlation between IAA internal exposure levels and reproductive outcomes. Secondly, the sensitivity and reliability of methods for detecting the internal exposure level of IAA in organisms should be further improved and optimized. It was not until May 2024 that a latest work reported that the modified QuEChERS sample preparation combined with gas chromatography-tandem triple quadrupole mass spectrometry (GC–MS/MS) could successfully detect the internal exposure levels of IAA in various biological samples (plasma, urine, feces, liver, kidney, and other tissues) (27). With the further verification and application of the improved method, it is expected that more population studies on the internal exposure level of IAA will appear soon. Thirdly, most current toxicological studies are descriptive and cross-sectional, lacking in-depth mechanism dissection, which increases the difficulty of targeted intervention and prevention. Hence, mechanism-based exploration should be further advanced and deepened by adopting new techniques and methods like multi-omics, which is employed in reproductive research to better understand the cellular-level molecular mechanism related to gametes and the role of reproduction-related proteins (52). Taken together, this work preliminarily sheds light on the harmful effects of IAA on reproductive health, but we still have more work to do in the face of this emerging disinfection byproduct, IAA.
